# MicroRNA-140-5p targets insulin like growth factor 2 mRNA binding protein 1 (IGF2BP1) to suppress cervical cancer growth and metastasis

**DOI:** 10.18632/oncotarget.11722

**Published:** 2016-08-31

**Authors:** Yanlin Su, Jie Xiong, Jinyue Hu, Xin Wei, Xuelian Zhang, Lijuan Rao

**Affiliations:** ^1^ Department of Obstetrics and Gynecology, Changsha Central Hospital, Changsha, China; ^2^ Department of Epidemiology and Health Statistcs, XiangYa School of Public Health, Central South University, Changsha, China; ^3^ Medical Research Center, Changsha Central Hospital, Changsha, China

**Keywords:** cervical cancer, microRNA-140-5p, IGF2BP1, tumor suppressor

## Abstract

MicroRNAs (miRNAs) are a class of small non-coding RNA molecules that play important roles in carcinogenesis and tumor progression. Previous studies have revealed that MicroRNA-140-5p (miR-140-5p) was abnormally expressed in several cancers. However, its function and possible mechanism in cervical cancer (CC) remains unknown. In this study, the data mining results showed that miR-140-5p was down-regulated in CC specimens and the down-regulation of miR-140-5p was associated with CC poor prognosis. These observations prompted us to further investigate the roles and mechanisms of miR-140-5p in human CC pathogenesis. We found that the over-expression/inhibition of miR-140-5p significantly decreased/increased cell proliferation, migration, and invasion in CC cells in vitro. Meanwhile, the results from in vivo assays showed that the over-expression of miR-140-5p induced significantly suppression of tumor growth and metastasis in nude mice. Furthermore, Insulin like growth factor 2 mRNA binding protein 1 (IGF2BP1) was identified as a direct target of miR-140-5p, and both gain-of-function and loss-of-function assays revealed that IGF2BP1 is also a functional target of miR-140-5p. Taken together, our findings suggested a novel miR-140-5p-IGF2BP1 regulatory circuit for CC pathogenesis, and miR-140-5p may be a potential target for CC therapy.

## INTRODUCTION

Cervical cancer (CC) is the third leading cause of cancer-related deaths among women worldwide, with an estimated 500,000 new cases and 300,000 deaths per year [1]. Although its mortality decreased along with advances in surgery, radiotherapy, and chemotherapy, patients with advanced CC still have very poor prognosis and significantly variable clinical outcomes due to tumor recurrence and metastasis. Therefore, it is important to uncover the molecular mechanisms of CC and identify molecular biomarkers which are of value in the developments of improved therapeutic strategies and prognosis.

MicroRNAs (miRNAs) are small, non-coding RNA molecules with 18–25 nucleotides. They inhibit mRNA translation and/or negatively regulate its stability by binding to the 3′-untranslated region (3′UTR) of target mRNAs [2]. Many miRNAs have been demonstrated as key regulators of many cellular biological processes such as development, proliferation, differentiation, and apoptosis [3]. In human cancers, there is growing evidence that miRNAs are involved in cancer cell proliferation, differentiation, metastasis and apoptosis by regulating oncogenes and/or tumor suppressor genes [4–10]. In CC, multiple miRNAs, such as miR-133b, miR-7, miR-20a, miR-10a, miR19, and miR-125b have been demonstrated to be involved in CC tumorigenesis and/or progression [11–16].

Our data-mining based on public available CC genomic data showed that miR-140-5p was down-regulated in CC samples and negatively correlated with poor prognosis of CC patients. These results prompt us to hypothesize that miR-140-5p may play a tumor-suppressor role in CC cells. The biological function of miR-140-5p has been studied only in a limited number of malignant carcinomas such as breast cancer, osteosarcoma, colon cancer, hepatocellular carcinoma and non-small cell lung cancer, and all these previous findings suggested that miR-140-5p functions as a tumor-suppressor in these cancers [17–21]. However, to our knowledge, its roles and the potential mechanisms in CC remain unclear.

In this study, we objected to illustrate the tumor-suppressor role of miR-140-5p in CC and elucidate the underlying molecular mechanism. We found that miR-140-5p was down-regulated in CC samples and cell lines, and was inversely correlated with the levels of insulin like growth factor 2 mRNA binding protein 1 (IGF2BP1) in CC patients. Cellular and model animal experiments revealed that miR-140-5p acted as a tumor suppressor by affecting CC cell proliferation, migration, and invasion. Furthermore, IGF2BP1 was identified as a direct functional target of miR-140-5p in CC, and it might exert its biological function by interaction with c-myc. Our findings described a novel role of miR-140-5p as a tumor suppressor by targeting IGF2BP1 in CC.

## RESULTS

### Down-regulation of miR-140-5p is associated with CC poor prognosis

**Figure 1:**
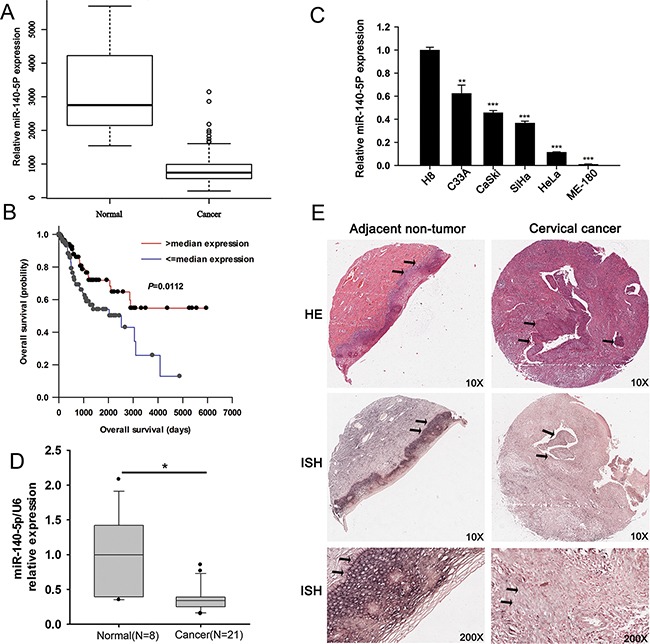
miR-140-5p is down-regulated in CCs and positively correlated with patient survival **A.** TCGA database analysis of miR-140-5p expression in CCs (n = 307) and in normal human cervix (n = 3). **B.** Survival analysis of miR-140-5p expression in TCGA database. **C.** qRT-PCR analysis of miR-140-5p levels in one normal cervical cell line (H8) and five CC cell lines (C33A, CaSki, SiHa, HeLa, and ME-180). **D.** qRT-PCR analysis of miR-140-5p levels in 8 normal cervix tissues and 21 CC tissues. **E.** Representative HE straining and in situ hybridization (ISH) analysis of miR-140-5p expression in CC tissues and in matched adjacent non-tumor specimens (n = 31).

We analyzed TCGA data for miR-140-5p levels and for correlation with patient survival. The results showed that miR-140-5p was significantly decreased in CC samples compared with that in normal cervical tissues (Figure 1A, *P* < 0.001). Univariate Cox regression analysis of 307 CC samples revealed that decreased miR-140-5p expression was a highly significant negative risk factor (HR = 0.89, *P* < 0.05). By dividing CC patients into high and low expression of miR-140-5p according to the median level of miR-140-5p, survival analysis revealed that the median survival time was significantly longer in CC patients with higher miR-140-5p expression than that in those with lower miR-140-5p expression (Figure 1B).

We also measured miR-140-5p levels in CC cell lines (C33A, HeLa, CaSki, SiHa, and ME-180) and normal human cervical epithelial cell line (H8), in human CC tissues (n = 21) and unmatched normal cervixes (n=8), and in human tumor (n = 31) and paired adjacent non-tumor cervical specimens. qRT-PCR results of cell lines showed that compared with that in H8, miR-140-5p was down-regulated with different expression levels in C33A (0.61 ± 0.14 fold), HeLa (0.13 ± 0.01 fold), SiHa (0.35 ± 0.05 fold), CaSki (0.43 ± 0.04 fold) and ME-180 (0.02 ± 0.003 fold) (Figure 1C). Similarly, qRT-PCR results of tissues revealed that miR-140-5p was significantly down-regulated in human CC tissues (n=21) compared with normal cervixes (n=8) (Figure 1D). Furthermore, in situ hybridization assay also confirmed an overt decrease of miR-140-5p in CC tissues (10 positive samples out of 31 samples) compared with that in their matched non-tumor adjacent tissues (19 positive samples out of 31 samples) (Figure 1D, *P* < 0.05).

In addition, we compared clinicopathological variables between CC patients with positive (n=10) and negative (21) miR-140-5p expressions, and found that a remarkably negative miR-140-5p expression was significantly associated with lymph node metastasis (Table 1).

**Table 1:** Correlation between clinicopathological variables and miR-140-5p expression in 31 CC patients

Variables	MiR-140-5p expression		P value
	Positive	Negative	
**Age**			
≥55 years	2	9	0.2617
<55 years	8	12	
**TNM**			
I-II	8	13	0.4285
III-IV	2	8	
**Tumor diameter**			
≥2 cm	8	19	0.5773
<2 cm	2	2	
**Lymph node metastasis**			
Yes	0	8	0.03171
No	10	13	

Fisher's exact test was used for ratio comparison due to sample size (n=31) < 40.

These data mining and expression analysis results prompted us to hypothesize that miR-140-5p may be involved in the tumorigenesis of CC.

### miR-140-5p suppresses CC cell proliferation in vitro

**Figure 2:**
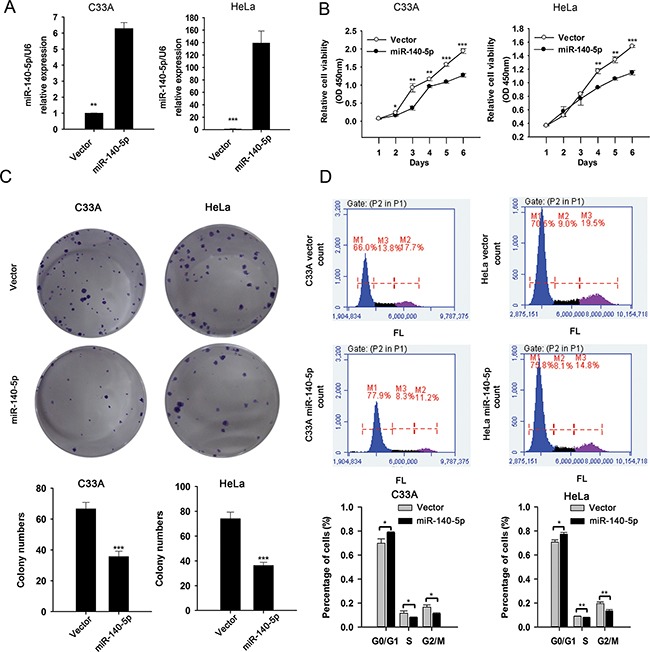
miR-140-5p over-expression suppresses CC cell proliferation in vitro. **A.** qRT-PCR analysis of miR-140-5p expression in miR-140-5p-transfected and mock-transfected cells. **B.** CCK8 assay of the effect of miR-140-5p over-expression on cell viability. **C.** The effect of miR-140-5p over-expression on the colony formation. **D.** PI staining and FACS analysis of the effect of miR-140-5p over-expression on cell cycle progress.

To explore the possible biological significance of miR-140-5p in CC cells, we constructed stable cell lines expressing miR-140-5p from C33A and HeLa cells (Figure 2A). CCK8 assays showed that the cell viability of C33A and HeLa cells stably transfected with miR-140-5p plasmid were significantly decreased compared with that of vector control cells (Figure 2B). In addition, the results from colony formation assay showed that over-expression of miR-140-5p significantly suppressed the potential of cell colony formation in both C33A and HeLa cells (Figure 2C). To further confirm the proliferation suppression function of miR-140-5p on cervical cancer cells, we investigated the cell cycle progress of C33A and HeLa cells by flow cytometry. As expected, miR-140-5p over-expression triggered an cell cycle arrest at G0/G1 phase, and the cell proportions at S and G2/M phases in both C33A and HeLa were deceased significantly (Figure 2D). The results indicated that miR-140-5p may suppress cell proliferation by inducing cell cycle arrest in CC. In parallel, we transfected C33A and HeLa cells with a miRNA inhibitor to inhibit expression of miR-140-5p (Supplementary Figure S1A). CCK8 and colony formation assays revealed that inhibition of miR-140-5p significantly increased cell viability (Supplementary Figure S1B) and colony formation (Supplementary Figure S1C) of C33A and HeLa cells.

In addition, we investigated whether miR-140-5p has any effect on CC cell death. miR-140-5p-transfected C33A and HeLa cells were stained with propidium iodide (PI), and analyzed by fluorescence microscope. The results showed that miR-140-5p over-expression was found to promote cell death in both C33A and HeLa cells significantly (Supplementary Figure S2).

### miR-140-5p inhibits CC cell migration and invasion in vitro

**Figure 3:**
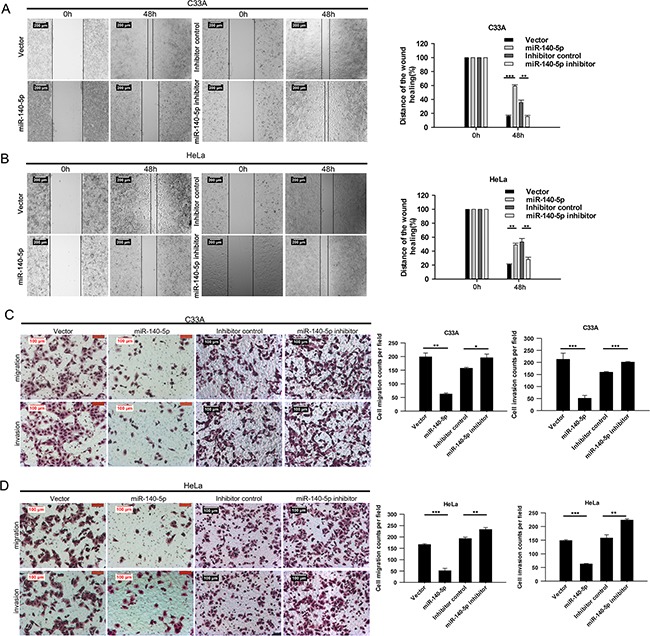
miR-140-5p negatively regulated the migration and invasion of CC cells in vitro Wound healing assays for miR-140-5p over-expression and inhibition in C33A **A.** and HeLa **B.** cells. Transwell migration and matrigel invasion assays for miR-140-5p over-expression and inhibition in C33A **C.** and HeLa **D.** cells.

To further illustrate the biological significance of miR-140-5p in CC cells, we investigated whether miR-140-5p could also inhibit cell migration and invasion in CC. The cell migration abilities of miR-140-5p-transfected and miR-140-5p inhibitor-transfected C33A and HeLa cells were measured by transwell migration and wound healing assays. Wound healing assay showed that the over-expression/inhibitation of miR-140-5p significantly decreased/increased would healing in both C33A (Figure 3A) and HeLa (Figure 3B) cells, and transwell migration assay revealed that the over-expression/inhibitation of miR-140-5p significantly decreased/increased cell migration in both C33A and HeLa cells (Figure 3C and 3D).

The cell invasion abilities of miR-140-5p-transfected and miR-140-5p inhibitor-transfected C33A and HeLa cells were measured by transwell invasion assays. Results showed that miR-140-5p transfection/inhibitation dramatically decreased/increased the cells invaded across the film (Figure 3C and 3D). These data suggested that miR-140-5p suppresses CC cell migration and invasion in vitro.

### miR-140-5p decreases CC growth and metastasis in vivo

**Figure 4:**
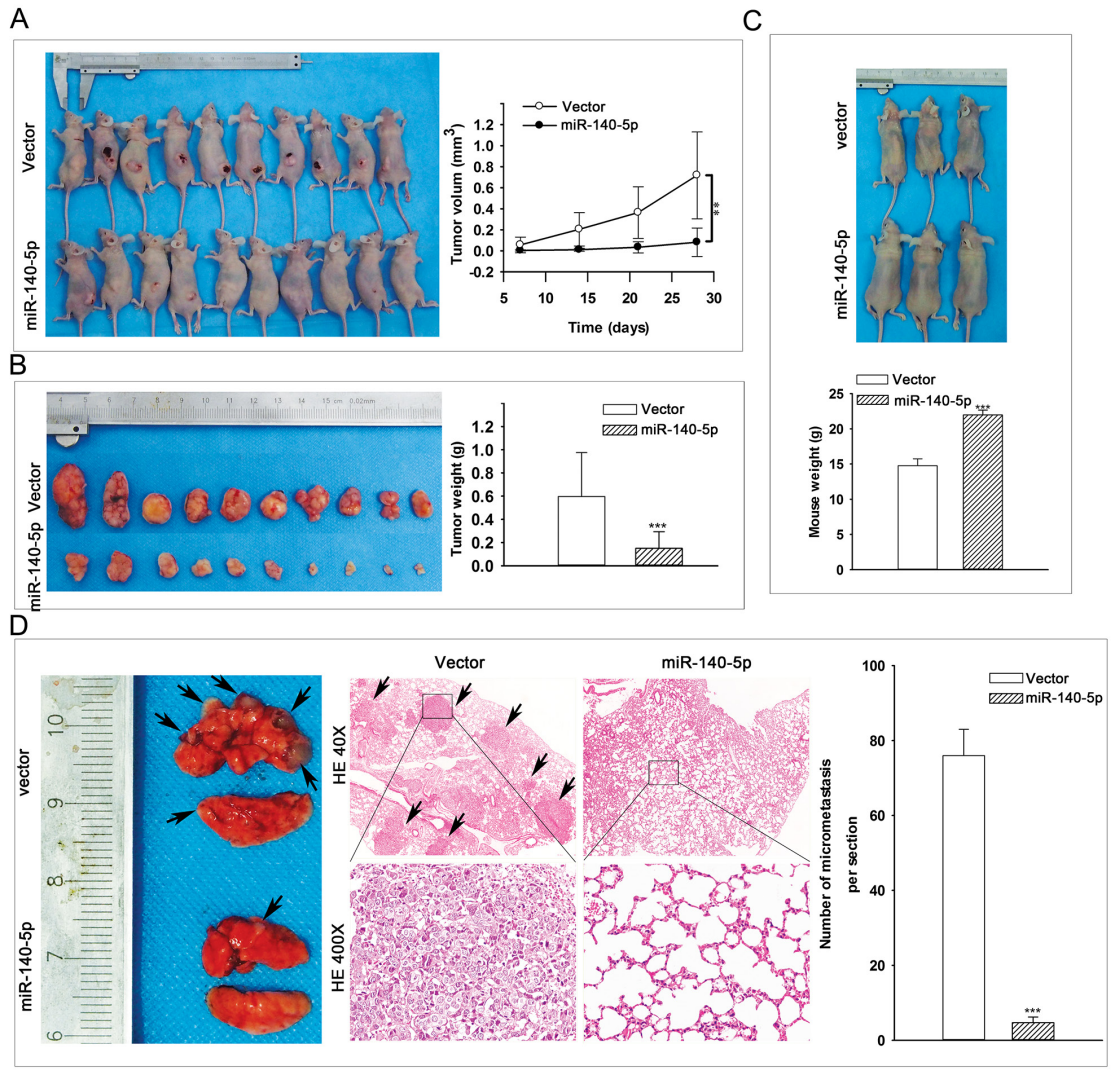
miR-140-5p over-expression suppresses CC tumor growth and metastasis in vivo **A-B.** In vivo nude mouse model analysis of the effect of miR-140-5p on tumor growth (injection by s.c.). **C.** In vivo nude mouse model analysis of the effect of miR-140-5p on mouse weight implanted with C33A (injection by caudal vein). **D.** Histological analysis of the effect of miR-140-5p on the tumor growth in nude mouse lung metastatic model.

We have observed the suppression effects of miR-140-5p on proliferation, migration, and invasion of CC cells in vitro, we then examined the role of miR-140-5p on CC tumor growth and metastasis in vivo. For the comparison of tumor growth, C33A-miR-140-5p and C33A-vector cells were inoculated subcutaneously into the flank of nude mice with 1 × 10^7^cells per mouse. The animals were closely monitored for tumor growth for 4 weeks, and tumor sizes were measured every 7 days. The results showed that the tumors from miR-140-5p over-expressing cells were significantly smaller in tumor volume and weight than that from control cells (Figure 4A and 4B). For comparison of tumor metastasis, C33A-miR-140-5p and C33A-vector cells were injected into nude mice via tail vein injection. The mice were sacrificed 8 weeks after cell injection, and their lungs were excised to observe metastatic nidi on the surface. The weights of mice inoculated with C33A-miR-140-5p cells were significantly heavier than that of mice inoculated with C33A-vector cells (Figure 4C), and the number of lung metastasis nodules was significantly decreased in C33A-miR-140-5p group compared with that in C33A-vector group (Figure 4D). Lung histological results showed that more cancer cells were observed in C33A-vector group than that in C33A-miR-140-5p group (Figure 4D). Taken together, these results indicated that miR-140-5p over-expression can suppress tumor growth and metastasis in vivo.

### miR-140-5p directly targets and down-regulates IGF2BP1

**Figure 5:**
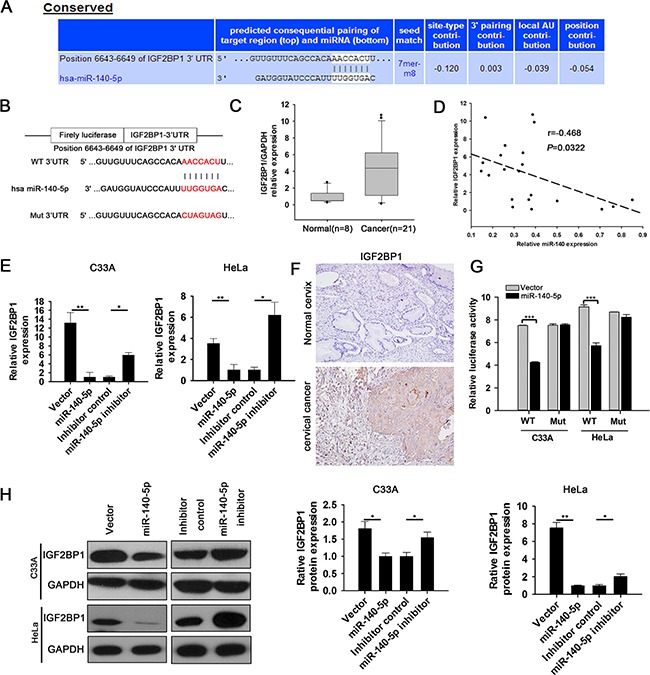
miR-140-5p directly targets IGF2BP1 in CC cells **A.** Targetscan program predicts that IGF2BP1 is a direct target gene of miR-140-5p. **B.** The wild-type (WT) or mutant (MUT) binding sequences of miR-140-5p on IGF2BP1 3′-UTR. **C.** qRT-PCR analysis of IGF2BP1 mRNA levels in 21 CC tissues and 8 Normal cervix tissues. **D.** Spearman's correlation analysis between miR140-5p levels and IGF2BP1 mRNA levels in 21 CC tissues. **E.** qRT-PCR analysis of the effects of miR-140-5p over-expression and inhibitor on IGF2BP1 mRNA levels in C33A and HeLa cells. **F.** IHC analysis of IGF2BP1 protein levels in normal cervix tissues and CC tissues. **G.** 3′UTR luciferase assay analysis of the capacity of miR-140-5p binding to the 3′UTR of IGF2BP1. **H.** Western blot analysis of the effects of miR-140-5p over-expression and inhibition on IGF2BP1 protein levels in C33A and HeLa cells.

To understand how miR-140-5p inhibits CC growth and metastasis, we searched potential targets of miR-140-5p by three computational algorithms (TargetScan, miRanda, and Pictar). Of the potential targets that were predicted by all three algorithms, insulin like growth factor 2 mRNA binding protein 1 (IGF2BP1) was identified as a candidate target of miR-140-5p (Figure 5A and 5B) and attracted our attention as it has been reported to be implicated in tumorigenesis [22]. We then measured IGF2BP1 mRNA levels in human CC tissues and cells (C33A, HeLa, CaSki, SiHa, and ME-180) as well as human normal cervixes and normal human cervical epithelial cell line (H8). As expected, IGF2BP1 mRNA levels were up-regulated in both human CC tissues (Figure 5C) and cell lines (Supplementary Figure S3A) compared with human normal cervixes and H8, respectively. Recalling the expression levels of miR-140-5p in human CC tissues and cell lines, a statistically significant inverse correlation was observed by Spearman's correlation analysis between RNA levels of miR-140-5p and mRNA levels of IGF2BP1 in both human CC tissues (Figure 5D) and cell lines (Supplementary Figure S3B). Furthermore, qRT-PCR and western blot assays revealed that over-expression/inibition of miR-140-5p significantly decreased/increased the expression of IGF2BP1 mRNA (Figure 5E) and protein (Figure 5H) levels in both C33A and HeLa cells, and IHC analysis revealed that IGF2BP1 was increased in human CC tissues (17 positive samples out of 21 samples) compared with that in human normal cervixes (2 positive samples out of 8 samples) (Figure 5F, *P*<0.01). These results prompted us to validate whether IGF2BP1 is the direct downstream target of miR-140-5p.

We cloned IGF2BP1 3′ UTR containing putative miR-140-5p binding site into a luciferase reporter vector. Luciferase reporter assay results revealed that miR-140-5p directly bound to IGF2BP1 3′ UTR by which it remarkably decreased the relative luciferase activity of IGF2BP1-3′UTR in C33A and HeLa cells, but had no effect on the mutant of IGF2BP1-3′UTR (Figure 5G). Taken together, these results indicated that IGF2BP1 is a direct downstream target of miR-140-5p in CC cells.

### IGF2BP1 is involved in miR-140-5p-induced CC suppression

**Figure 6:**
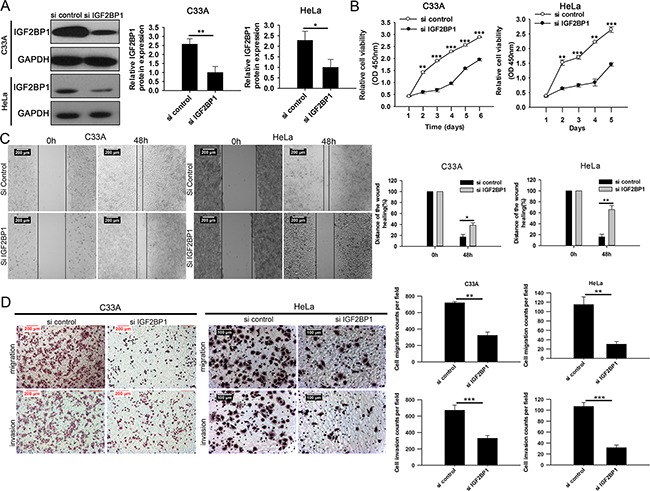
IGF2BP1 interference decreased CC cell proliferation, migration and invasion in vitro **A.** Western blot analysis of IGF2BP1 expression in IGF2BP1-siRNA-transfectd or mock-transfected C33A and HeLa cells. **B.** CCK8 assays analysis of the effects of siIGF2BP1 on cell proliferation in C33A and HeLa cells. **C.** Wound healing assays analysis of the effects of siIGF2BP1 on cell migration in C33A and HeLa cells. **D.** Transwell assays analysis of the effects of siIGF2BP1 on cell invasion and migration in C33A and HeLa cells.

To investigate whether the tumor suppressor function of miR-140-5p is mediated by IGF2BP1, C33A and HeLa cells were stably transfected with IGF2BP1 siRNA and IGF2BP1. Western blotting analysis confirmed that the expression of IGF2BP1 was significantly suppressed (Figure 6A) and increased (Supplementary Figure S4A) in both cells, respectively. Cellular function assays revealed that siIGF2BP1 significantly decreased cell viability (Figure 6B), migration (Figure 6C and 6D) and invasion (Figure 6D) in C33A and HeLa cells, and IGF2BP1 over-expression led to opposite effects on cell viability (Supplementary Figure S4B), migration (Supplementary Figure S4C and S4D) and invasion (Supplementary Figure S4D). These results indicated that the effects of IGF2BP1 RNA interference/over-expression were similar to that of miR-140-5p over-expression/inhibition.

**Figure 7:**
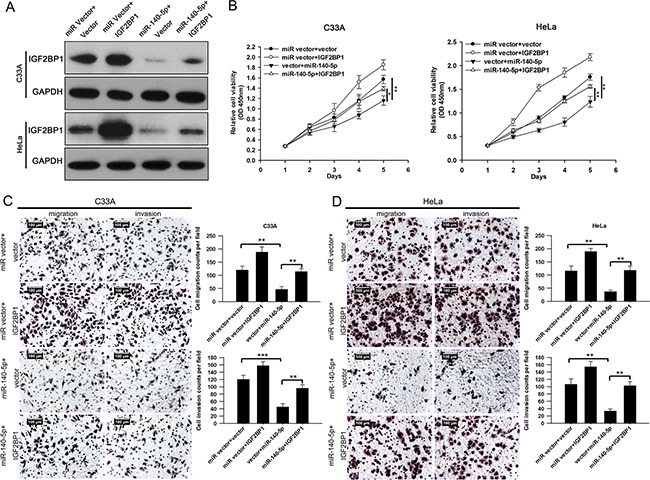
IGF2BP1 is involved in miR-140-5p-induced proliferation, migration and invasion inhibition in C33A and HeLa cells C33A and HeLa cells were transfected with specific miR vector+vector, miR vector+IGF2BP1, miR-140-5p+vector or transfected with IGF2BP1 plasmid lacking 3′UTR along with miR-140-5p. **A.** Western blotting analysis. **B.** cell viability assay (CCK-8). **C-D.** Transwell migration and invasion assays.

Furthermore, miR-140-5p transfected C33A and HeLa cells were transfected with IGF2BP1 plasmids lacking 3′UTR (Figure 7A). Cellular function assays showed that over-expression of IGF2BP1 significantly rescued miR-140-5p induced inhibition of cell proliferation (Figure 7B), migration and invasion (Figure 7C and 7D) in C33A and HeLa cells. Taken together, these data suggested that IGF2BP1 is involved in miR-140-5p-induced CC suppression, and it is functional target of miR-140-5p.

## DISCUSSION

miRNAs have been reported to be dysregulated in many types of cancers, and they can contribute to carcinogenesis and progression of cancers [23] as either tumor suppressors or promoters [24]. miR-140-5p has been investigated in breast cancer [17], osteosarcoma and colon cancer [18], hepatocellular carcinoma [19,20], and non-small cell lung cancer [21]. All these previous findings suggest that miR-140-5p functions as a tumor-suppressor. In breast cancer, miR-140-5p has been identified as a tumor suppressor due to the interaction with SOX2 [17]. In addition, it has been suggested that miR-140-5p plays a role in the development of chemoresistance in human osteosarcoma and colon cancers by reduced cell proliferation through G1 and G2 phase arrest mediated in part through the suppression of HDAC4 [18]. miR-140-5p also plays a tumor suppressor role in hepatocellular carcinoma by controlling NF-κB activity by directly regulating DNMT1 expression [19], and suppresses tumor growth and metastasis by targeting TGFBR1 and FGF9 [20]. miR-140-5p expression is also reduced in non-small cell lung cancer, and it suppresses tumor growth and metastasis by targeting IGF1R [21]. However, the roles and potential mechanisms of miR-140-5p in CC remain unclear. In this study, we revealed that miR-140-5p is commonly down-regulated in CC, and in vitro and in vivo assays further demonstrated that miR-140-5p suppresses CC cell proliferation, migration and invasion by direct targeting IGF2BP1.

IGF2BP1, also known as IMP-1 or CRD-BP, is a member of the RNA-binding proteins (RBPs) [25] which exert their function to regulate the localization, stability, or translation of their target RNAs by direct interaction [26]. IGF2BP1 belongs to a conserved family of RNA-binding, oncofetal proteins, which includes IGF2BP2 and IGF2BP3 [22]. Several target mRNAs of IGF2BP1 were identified. For example, IGF2BP1 controls the transport and translation of ACTB mRNA in developing axons and dendrites by direct interaction [27–30]. Interaction of IGF2BP1 with c-MYC mRNA inhibits CRD-dependent mRNA decay and is correlated with poor prognosis in ovarian carcinoma [31–33].

Previous studies about IGF2BP1 in cancers mainly focus on its expression and/or correlations between its expression and clinical variables [34]. Until recently, functional studies have emerged to find out the underling mechanism of IGF2BP1 involved in cancers. For example, IGF2BP1 is involved in liver carcinogenesis by binding to and stabilizing the c-MYC and MKI67 mRNAs, and it is an important pro-tumorigenic factor for hepatocellular carcinoma [35]. miR-625 suppresses hepatocellular carcinoma migration and invasion by targeting IGF2BP1 and further affects the IGF2BP1/PTEN pathway [36]. However, neither the regulation nor the specific functional role of IGF2BP1 in CC has been studied. In the present study, we showed that IGF2BP1 is commonly up-regulated both in human CC tissues and cell lines. IGF2BP1 is a direct functional target of miR-140-5p, and is involved in the miR-140-5p induced suppression of CC development. Our study also provided a new mechanism of IGF2BP1 down-regulation in CC. We further hypothesized whether IGF2BP1 exerts its function by targeting c-MYC mRNA in CC. IHC analysis revealed that c-MYC was increased in human CC tissues compared with that in human normal cervix (Supplementary Figure S5A), and western blot analysis showed that both the over-expression of miR-140-5p and IGF2BP1 RNA interference in C33A similarly decreased the protein expression of c-MYC (Supplementary Figure S5B). Although these preliminary results illuminated our further research direction, more direct evidences are needed to support the hypothesis.

In this study, miR-140 was constructed as over-expression vector, and miR-140 may express both miR-140-5p and miR-140-3p. We checked the expression of miR-140-3p in CC tissues (n=21) and unmatched normal cervixes (n=8), and in CC cells transfected with over-expression vector by qRT-PCR. Results showed that miR-140-3p was also down-regulated in CC tissues compared with normal cervixes and significantly amplified in CC cells transfected with over-expression vector. Thus the in vitro assays of CC cells transfected with over-expression vector demonstrated that both miR-140-5p and miR-140-3p may act as CC suppressor. However, the subsequent inhibition assays, luciferase reporter assay, and rescue assays further demonstrated that miR-140-5p exerts its tumor suppressor role in CC cells by targeting IGF2BP1. For miR-140-3p, we do not investigate its functions in CC cells by assays. However, miR-140-3p has been reported to be involved in lung cancer suppression [37–38] and diagnosis of lung squamous cell carcinoma [39]. Thus though the role of miR-140-3p in CC cells is still unclear, we speculate that miR-140-3p may acts as a tumor suppressor in CC cells, and investigations will be expanded on this issue in the future.

In conclusion, this study identified a novel miR-140-5p-IGF2BP1 axis which accounts for CC pathogenesis and has potential therapeutic value for CC treatment.

## MATERIALS AND METHODS

### The cancer genome atlas (TCGA) data analysis

Clinical and level 3 miRNA sequencing data for 307 CC patients and 3 normal unmatched cervical samples were obtained from TCGA data portal (http://tcga-data.nci.nih.gov/tcga/) in March 2014. The data collection was compliant with all laws and regulations for the protection of human subjects, and necessary ethical approvals were obtained. Batch effects were removed by *Combat* function incorporated in *sva* R package [40]. The R *Limma* package [41] was used to compare the expression of miR-140-5p between CC samples and normal cervical samples. The relationship between miR-140-5p expression and the survival of CC patients was assessed by univariate Cox regression [42]. Kaplan-Meier method [43] was used to analyze the survival difference between the high miR-140-5p expression CC patients and the low miR-140-5p expression CC patients.

### Tumor specimens and cell lines

CC tissue samples (n = 21) and unmatched normal cervical epithelium samples (n = 8) were collected from the Department of Gynecologic Oncology of Changsha Central Hospital from 2012 to 2014. No previous systemic or local treatment had been conducted on these patients before the operation or biopsy. Informed consent was obtained from all patients, and the study was approved by the ethics committee of Changsha Central Hospital. The tissues were frozen in liquid nitrogen immediately after surgical removal and stored at −80°C until use. Five CC cell lines (C33A, HeLa, CaSki, SiHa, ME-180) and a normal human cervical epithelial cell line (H8) were kindly provided by professor Guancheng Li from Cancer Research Institute, Central South University (Changsha, China). These cells were maintained at 37°C in an atmosphere of 5% CO_2_ in DMEM with 10% fetal bovine serum.

### Quantitative real-time reverse transcription -polymerase chain reaction (qRT-PCR)

TRIzol reagent (Invitrogen) was used to extract RNA from tissues and cell lines according to the manufacturer's instructions. RNAs were reverse transcribed using the RevertAid First Strand cDNA Synthesis Kit (Thermo fisher, USA). U6 and GAPDH were used as internal control, and the primers were synthesized (Sangon, Shanghai, China) as follows:

IGF2BP1-F: CAGGAGATGGTGCAGGTGTTTATCC;
IGF2BP1-R: GTTTGCCATAGATTCTTCCCTGAGC;
c-Myc-F: CAGCTGCTTAGACGCTGGATT;
c-Myc-R: GTAGAAATACGGCTGCACCGA;
GAPDH-F: AGACACCATGGGGAAGGTGAA;
GAPDH-R: ATTGCTGATGATCTTGAGGCTG.

Primers of miR-140-5p and U6, and related miRNA reagents were purchased from RiboBio Co., Ltd (miRQ0000573-1-2, MQP-0202) (Guangzhou, China). The reaction conditions were 40 amplification cycles of 95°C for 3 min, 95°C for 12 s, and 62°C for 50 s using the LightCycler 96 Real-Time PCR System (Roche, Switzerland) with the SYBR Green Real time Master Mix (Roche, Switzerland). Each sample was analyzed in triplicate. Comparative threshold cycle method-fold change (2^−ΔΔCT^) was used to analyze relative changes.

### In situ hybridization (ISH) for miRNA-140-5p

Commercial TMAs were used to analyze the expression of has-miR-140-5p in CC tissues based on ISH analysis. TMA consisted of 31 cervical squamous tissues and matched paraneoplastic tissues (catalog no. OD-CT-RpUtr03-003, Shanghai Outdo Biotech, China). Locked nucleic acid (LNA)-modified oligonucleotide probes (Exiqon) were used for ISH [44]. The sections were firstly deparaffinized by xylene 3 times for 5 min each time. After being washed in 100% ethanol (5 min), 70% ethanol (5 min), 50% ethanol (5 min), 25% ethanol (5 min), DEPC-treated water (1 min) and PBS (twice, 5 min each time) individually, the sections were deproteinated by the digestion of proteinase K (3 μg/ml) at 37°C for 1 h. Then the sections were washed in 0.2% glycine for 30 s, in PBS twice for 30 s each time and were fixed with 10% formalin for 10 min. After being rinsed 2 times in PBS, the sections were covered by hybridization buffer (50% formamide, 5× SSC, 0.1% Tween, 9.2 mM citric acid for adjustment to pH6, 50 μg/ml heparin, 500 μg/ml yeast RNA) and incubated at room temperature for 2 h. Then the sections were added with 200 μl hybridization mix (hybridization buffer with 20 nM miRNA detection probe), covered by Nescofilms and incubated in a humidified chamber at room temperature overnight. Then the sections were rinsed twice in 2 × SSC (Invitrogen) at 37°C, 3 times in 50% formamide, 2× SSC at room temperature for 30 min and 5 times in PBST (PBS+0.1%Tween20) at room temperature for 5 min. After 1 h in blocking buffer (2 mg/ml BSA in PBST) at room temperature, the sections were incubated with antibody (1:1000 anti-DIG-AP Fab fragments in Antibody Diluent (Zymed, Cat. No. 003218)) in a humidified chamber at 4°C over-night. Subsequently, the sections were washed 5 times in PBST for 5 min and 3 times in TBS for 5 min. Then the color of LNA-ISH was developed by the light sensitive color reaction solution (1 ml solution B + 3.3 μl 50 mg/ml BCIP + 4.4 μl NBT) at 37°C in a humidified chamber for 75 min. Then the sections were washed 3 times in PBST for 5 min, placed in room temperature to air dry, and mounted by Histomount. The photos of the LNA-ISH were taken under the microscope (Leica).

### Vector construction and transfection

cDNAs encoding hsa-miR-140 sequences were subcloned into a pEZX-MR03 puro DNA plasmid (GeneCopoeia, MD, USA) in which a puromycin selectable marker was encoded. cDNAs encoding IGF2BP1 sequences were subcloned into a EX-NEG-M98 DNA plasmid (GeneCopoeia, MD, USA) in which a Neomycin selectable marker was encoded. All cloned fragments were verified by sequencing. HeLa and C33A cells were transfected with hsa-miR-140 plasmid, mock plasmid (negative control), IGF2BP1 plasmid, mock plasmid (negative control), miRNA Inhibitor, Inhibitor control (miR20000431-1-5, RiboBio, Guangzhou, China) using Lipofectamine 2000 (Invitrogen, Carlsbad, CA, USA) in accordance with the manufacturer's instructions. Cells transfected with plasmid were then cultured with medium containing puromycin (400 μg/ml) or G418(500 μg/ml). Colonies were chosen 21 days after transfection.

### Cell proliferation assay

Cell proliferation was measured using a Cell Counting Kit-8 (CCK-8, Dojindo Molecular Technologies, Japan). Cells were seeded into 96-well plates at 2000 cells per well. After incubation for a series of time periods, 10 μl CCK-8 was added to each well and incubated for 1 h at 37°C. Cell viability was determined by measuring the absorbance at 450 nm. All experiments were performed three times and were calculated using average results, which we used to draw the growth curves. Growth inhibition rate was calculated as following: (AC−AT)/AC×100% (AC = absorbance value of the control group, and AT = absorbance value of the experimental group).

### Cell migration and invasion assays

Cell migration and invasion were detected using Transwell chambers (8 μm pore size; Millipore) with(invasion assay) or without (migration assay) Matrigel(BD Biosciences, San Jose, CA, USA) matrix. In brief,600 μl complete medium was added to the bottom chamber, transfected cells were suspended in serum-free medium, and 200 μl of the cell suspension (containing 1×10^5^cells) was placed in the upper chamber. After 48 hours, the cells on the top surface of the membrane were mechanically removed using a cotton swab, and the cells on the bottom surface of the membrane were fixed in 95% ethanol and stained with 0.2% crystal violet solution. Cells adhering to the bottom surface of the membrane were counted in five randomly selected areas under a 200× microscope field. Each experiment was repeated three times.

### Wound healing assays

To further investigate the cell migration, we used wound healing assay. 8×10^5^ cells were seeded in each well of a 6-well plate. After 24h, when the well was almost full of cells, confluent cells were incubated in the presence of 20 μM mitomycin C for 2 h to inhibit cell proliferation, the cell monolayer was scraped straightly by using a micropipette tip and washed with phosphate-buffered saline (PBS) to remove cell debris. The scraped monolayer was incubated in serum-free medium for 48 h, and gap distances at indicated time points after wounding were measured under a light microscope(Laica, Germany). Image J Plus was used to quantify the wound healing assays. Each experiment was repeated three times.

### Cell colony formation assay

500 cells were placed in 6-well plate and maintained in DMEM containing 10% FBS for 2 weeks. Colonies were fixed with methanol and stained with 0.1% crystal violet in 20% methanol for 15 min. Cells were counted under microscope (Laica, Germany).

### Cell cycle assay

Cells were collected and fixed with 70% ethanol at 4°C for 24 h. After washing with PBS, cells were treated with RNase A (50 μg/ml) and stained with propidium iodide (25 μg/ml) for 30 min at 37°C. Samples were analyzed using a flow cytometer and distribution of cell-cycle phases was determined using Modfit Software (BD Biosciences, New Jersey, USA).

### PI staining of dead cell

The death of C33A and HeLa cells were detected by PI staining. In general, cells were cultured for 48 h. The culture supernatant was discarded and the cells were incubated in 1 mL PBS containing 0.5 μg/mL PI (Jamay. Beijing, China) and 100 μg/mL RNase A at RT for 30 min. The dried cells were shown with red fluorescence by fluorescence microscope (Laica, Germany).

### In vivo tumor xenograft studies and metastasis assays

Twenty 6-week-old female BALB/c nude mice were purchased from SLAC laboratory animal company (Shanghai, China), and maintained under pathogen-free conditions. In our experiments, 1 × 10^7^ cells in 0.2 mL PBS was subcutaneously injected into the flank of mice, leading to the formation of a tumor per animal. Tumor diameters were measured every 7 days. Tumor growth was monitored by the tumor volume, which was calculated as described: volume (mm^3^) = width^2^ (mm^2^) × length (mm) × 1/2. For in vivo metastasis assays, 2 × 10^6^ cells were injected into the caudal vein of nude mice (3 per group). After 8 weeks, the mice were killed, and lung metastatic colonization was monitored and quantified.

### Western blot analysis

Cellular proteins were prepared using cell lysis buffer (50 mM Tris-HCl, pH 8.0, 1% NP-40, 2 mM EDTA, 10 mM NaCl, 2 mg/ml aprotinin, 5 mg/ml leupeptin, 2 mg/ml pepstatin, 1 mM DTT, 0.1% SDS and 1 mM phenylmethylsulfonyl fluoride). Equal amounts of protein (50 μg) were separated by 10% SDS PAGE and then transferred to nitrocellulose membranes (Merck Millipore, Germany) by electroblotting. The membranes were blocked with 5% nonfat dry milk in TBST (10 mM Tris-HCl, pH 8.0, 150 mM NaCl, and 0.05% Tween 20) for 1 h, and then incubated with anti-IGF2BP1 (1:1000, Sigma-aldrich) or anti-c-Myc (1:1000, Sigma-aldrich) primary antibody overnight at 4°C before subsequent incubation with second antibody (Cell Signaling Technology, 1:1000) for 1 h at 37°C. Protein binds were visualized using enhanced chemiluminescence reagent (Pierce).

### Immunohistochemistry (IHC)

The sections from paraffin-embedded blocks were deparaffinized in xylene, then rehydrated in a graded ethanol series. Antigen retrieval was performed by microwaving for 3 minutes in citrate-buffered solution (pH = 6.0). Tissues were washed with PBS for 3 times for 3 minutes, and endogenous peroxidase activity was quenched by incubation for 20 minutes in 3% hydrogen peroxide. For the purpose of preventing nonspecific staining, the sections were preincubated with 10% goat serum at room temperature for 30 minutes. The sections were incubated with the following primary antibodies at 4°C overnight: anti-IGF2BP1 (1:100, Sigma-aldrich), anti- c-Myc (1:100, Sigma-aldrich). After being washed with PBS, a secondary goat anti-rabbit antibody conjugated with horseradish peroxidase was applied for 1 hour at room temperature. Finally, the sections were stained with 3, 3- diaminobenzidine tetrahydrochloride (DAB), and were then counterstained with hematoxylin.

### Small interfering RNA (siRNA) experiments

siRNA oligonucleotide against IGF2BP1 was purchased from Era-Biotech (Shanghai, China), with a scrambled siRNA serving as a control. Transfection was performed using Lipofectamine 2000 Transfection Reagent according to the manufacturer's protocol. Forty-eight hours after transfection, the cells were prepared for the appropriate downstream assay.

### Target genes prediction

Three online softwares, TargetScan (http://www.targetscan.org/vert_60/) [45], PicTar (http://pictar.mdc-berlin.de/) [46], and miRanda (http://www.microrna.org/) [47] were used to predict potential target genes of miR-140-5p.

### Luciferase reporter assay

miR-140-5p binding sites from 3′ UTR or mutant 3′ UTR of IGF2BP1were cloned into the pGL3 reporter luciferase vector (GeneChem). For reporter assay, 100 ng miR-140-5p vector or control miRNA was co-transfected with 0.1μg of the pGL3–3′UTR wild type or mutant plasmid DNAs into C33A cells in 96-well plates using Lipofectamine 2000. Luciferase activity in each well was quantified 24 hours after transfection using Dual- Luciferase Reporter Assay System (Promega, Madison, WI, USA) according to the manufacturer's protocol. The relative luciferase activity was determined by dividing the firefly luciferase activity by the pRL-TK luciferase activity.

### Statistical analysis for experimental data

All assays were repeated for three times and experimental data were analyzed and presented as mean ± SD. T-test was used for two groups comparisons when the data satisfy normal distribution, otherwise, an alternative non-parametric method (Mann-Whitney U test) was applied. Rate comparisons were conducted by chi-squared test or Fisher's exact test if appropriate. P values that less than 0.05 were considered statistically significant with representation *P < 0.05, **P<0.01, ***P<0.001.

## SUPPLEMENTARY MATERIALS FIGURES



## Abbreviations

CC: cervical cancer
miRNA: microRNA
qRT-PCR: quantitative RT-PCR
IGF2BP1: Insulin like growth factor 2 mRNA binding protein 1
3′UTR: 3′-untranslated region
siRNA: small interfering RNA
PI: propidium iodide
ISH: in situ hybridization
TCGA: The Cancer Genome Atlas
IHC: Immunohistochemistry
GAPDH: glyceraldehydes-3-phosphate dehydrogenase

## References

[1] Jemal A, Bray F, Center MM, Ferlay J, Ward E, Forman D (2011). Global cancer statistics. CA Cancer J Clin.

[2] Ambros V (2004). The functions of animal microRNAs. Nature.

[3] Bartel DP (2004). MicroRNAs: Genomics, biogenesis, mechanism, and function. Cell.

[4] Tavazoie SF, Alarcon C, Oskarsson T, Padua D, Wang Q, Bos PD, Gerald WL, Massagué J (2008). Endogenous human microRNAs that suppress breast cancer metastasis. Nature.

[5] Kefas B, Godlewski J, Comeau L, Li Y, Abounader R, Hawkinson M, Lee J, Fine H, Chiocca EA, Lawler S, Purow B (2008). microRNA-7 inhibits the epidermal growth factor receptor and the Akt pathway and is down-regulated in glioblastoma. Cancer Res.

[6] Razumilava N, Bronk SF, Smoot RL, Fingas CD, Werneburg NW, Roberts LR, Mott JL (2012). miR-25 targets TNF-related apoptosis inducing ligand (TRAIL) death receptor-4 and promotes apoptosis resistance in cholangiocarcinoma. Hepatology.

[7] Rothschild SI, Tschan MP, Federzoni EA, Jaggi R, Fey MF, Gugger M, Gautschi O (2012). MicroRNA-29b is involved in the Src-ID1 signaling pathway and is dysregulated in human lung adenocarcinoma. Oncogene.

[8] Zheng B, Liang L, Huang S, Zha R, Liu L, Jia D, Tian Q, Wang Q, Wang C, Long Z, Zhou Y, Cao X, Du C, Shi Y, He X (2012). MicroRNA-409 suppresses tumour cell invasion and metastasis by directly targeting radixin in gastric cancers. Oncogene.

[9] Zuo QF, Cao LY, Yu T, Gong L, Wang LN, Zhao YL, Xiao B, Zou QM (2015). MicroRNA-22 inhibits tumor growth and metastasis in gastric cancer by directly targeting MMP14 and Snail. Cell Death & Disease.

[10] Zuo QF, Zhang R, Li BS, Zhao YL, Zhuang Y, Yu T, Gong L, Li S, Xiao B, Zou QM (2015). MicroRNA-141 inhibits tumor growth and metastasis in gastric cancer by directly targeting transcriptional co-activator with PDZ-binding motif, TAZ. Cell Death & Disease.

[11] Qin W, Dong P, Ma C, Mitchelson K, Deng T, Zhang L, Sun Y, Feng X, Ding Y, Lu X, He J, Wen H, Cheng J (2011). MicroRNA-133b is a key promoter of cervical carcinoma development through the activation of the ERK and AKT1 pathways. Oncogene.

[12] Liu S, Zhang P, Chen Z, Liu M, Li X, Tang H (2013). MicroRNA-7 downregulates XIAP expression to suppress cell growth and promote apoptosis in cervical cancer cells. FEBS Letters.

[13] Kang HW, Fang W, Qian W, Zhao YF, Liu M, Li X, Tang H (2012). miR-20a promotes migration and invasion by regulating TNKS2 in human cervical cancer cells. FEBS Letters.

[14] Long MJ, Wu FX, Li P, Liu M, Li X, Tang H (2012). MicroRNA-10a targets CHL1 and promotes cell growth, migration and invasion in human cervical cancer cells. Cancer Letters.

[15] Xu XM, Wang XB, Chen MM, Tao L, Li YX, Jia WH, Liu M, Li X, Tang H (2012). MicroRNA-19a and -19b regulate cervical carcinoma cell proliferation and invasion by targeting CUL5. Cancer Letters.

[16] Cui F, Li X, Zhu X, Huang L, Huang Y, Mao C, Yan Q, Zhu J, Zhao W, Shi H (2012). MiR-125b inhibits tumor growth and promotes apoptosis of cervix cancer cells by targeting phosphoinositide 3-kinase catalytic subunit delta. Cellular Physiology and Biochemistry.

[17] Zhang Y, Eades G, Yao Y, Li Q, Zhou Q (2012). Estrogen receptor α signaling regulates breast tumor-initiating cells by down-regulating miR-140 which targets the transcription factor SOX2. J Biol Chem.

[18] Song B, Wang Y, Xi Y, Kudo K, Bruheim S, Botchkina GI, Gavin E, Wan Y, Formentini A, Kornmann M, Fodstad O, Ju J (2009). Mechanism of chemoresistance mediated by miR-140 in human osteosarcoma and colon cancer cells. Oncogene.

[19] Yang H, Fang F, Chang R, Yang L (2013). MicroRNA-140-5p suppresses tumor growth and metastasis by targeting transforming growth factor β receptor 1 and fibroblast growth factor 9 in hepatocellular carcinoma. Hepatology.

[20] Takata A, Otsuka M, Yoshikawa T, Kishikawa T, Hikiba Y, Obi S, Goto T, Kang YJ, Maeda S, Yoshida H, Omata M, Asahara H, Koike K (2013). MicroRNA-140 Acts as a Liver Tumor Suppressor by Controlling NF-jB Activity by Directly Targeting DNA Methyltransferase 1 (Dnmt1) Expression. Hepatology.

[21] Yuan Y, Shen Y, Xue L, Fan H (2013). MiR-140 suppresses tumor growth and metastasis of non-small lung cancer by targeting insulin-like growth factor 1 receptor. PloS One.

[22] Bell JL, Wächter K, Mühleck B, Pazaitis N, Köhn M, Lederer M, Hüttelmaier S (2013). Insulin-like growth factor 2 mRNA-binding proteins (IGF2BPs): post-transcriptional drivers of cancer progression?. Cell Mol Life Sci.

[23] Garzon R, Calin GA, Croce CM (2009). MicroRNAs in Cancer. Annual Review of Medicine.

[24] Esquela-Kerscher A, Slack FJ (2006). Oncomirs-microRNAs with a role in cancer. Nat Rev Cancer.

[25] Stohr N, Huttelmaier S (2012). IGF2BP1: a post-transcriptional “driver” of tumor cell migration. Cell Adh Migr.

[26] Mignone F, Gissi C, Liuni S, Pesole G (2002). Untranslated regions of mRNAs. Genome Biol.

[27] Leung KM, van Horck FP, Lin AC, Allison R, Standart N, Holt CE (2006). Asymmetrical beta-actin mRNA translation in growth cones mediates attractive turning to netrin-1. Nat Neurosci.

[28] Farina KL, Huttelmaier S, Musunuru K, Darnell R, Singer RH (2003). Two ZBP1 KH domains facilitate beta-actin mRNA localization, granule formation, and cytoskeletal attachment. J Cell Biol.

[29] Huttelmaier S, Zenklusen D, Lederer M, Dictenberg J, Lorenz M, Meng X, Bassell GJ, Condeelis J, Singer RH (2005). Spatial regulation of beta-actin translation by Src-dependent phosphorylation of ZBP1. Nature.

[30] Ross AF, Oleynikov Y, Kislauskis EH, Taneja KL, Singer RH (1997). Characterization of a beta-actin mRNA zipcode-binding protein. Mol Cell Biol.

[31] Weidensdorfer D, Stöhr N, Baude A, Lederer M, Köhn M, Schierhorn A, Buchmeier S, Wahle E, Hüttelmaier S (2009). Control of c-myc mRNA stability by IGF2BP1-associated cytoplasmic RNPs. RNA.

[32] Lemm I, Ross J (2002). Regulation of c-myc mRNA decay by translational pausing in a coding region instability determinant. Mol Cell Biol.

[33] Kobel M, Weidensdorfer D, Reinke C, Lederer M, Schmitt WD, Zeng K, Thomssen C, Hauptmann S, Huttelmaier S (2007). Expression of the RNA-binding protein IMP1 correlates with poor prognosis in ovarian carcinoma. Oncogene.

[34] Dimitriadis E, Trangas T, Milatos S, Foukas PG, Gioulbasanis I, Courtis N, Nielsen FC, Pandis N, Dafni U, Bardi G, Ioannidis P (2007). Expression of oncofetal RNA-binding protein CRD-BP/IMP1 predicts clinical outcome in colon cancer. Int J Cancer.

[35] Tony G, Hämmerle M, Pazaitis N, Bley N, Fiskin E, Uckelmann H, Heim A, Groβ M, Hofmann N, Geffers R, Skawran B, Longerich T, Breuhahn K, Schirmacher P, Mühleck B, Hüttelmaier S (2014). Insulin-Like Growth Factor 2 mRNA-Binding Protein 1 (IGF2BP1) Is an Important Protumorigenic Factor in Hepatocellular Carcinoma. Hepatology.

[36] Zhou X, Zhang CZ, Lu SX, Chen GG, Li LZ, Liu LL, Yi C, Fu J, Hu W, Wen JM, Yun JP (2015). miR-625 suppresses tumor migration and invasion by targeting IGF2BP1 in hepatocellular carcinoma. Oncogene.

[37] Kong XM, Zhang GH, Huo YK, Zhao XH, Cao DW, Guo SF, Li AM, Zhang XR (2015). MicroRNA-140-3p inhibits proliferation, migration and invasion of lung cancer cells by targeting ATP6AP2. International Journal of Clinical & Experimental Pathology.

[38] Wei D, Yao C, Teng X, Chai J, Yang X, Li B (2015). MiR-140-3p suppressed cell growth and invasion by downregulating the expression of ATP8A1 in non-small cell lung cancer. Tumor Biology.

[39] Tan X, Qin W, Zhang L, Hang J, Li B, Zhang C, Wan J, Zhou F, Shao K, Sun Y, Wu J, Zhang X, Qiu B, Li N, Shi S, Feng X (2011). A 5-microRNA signature for lung squamous cell carcinoma diagnosis and hsa-miR-31 for prognosis. Clinical Cancer Research.

[40] Johnson WE, Li C, Rabinovic A (2007). Adjusting batch effects in microarray data using empirical bayes methods. Biostatistics.

[41] Ritchie ME, Phipson B, Wu D, Hu Y, Law CW, Shi W, Smyth GK (2015). Limma powers differential expression analyses for RNA-sequencing and microarray studies. Nucleic Acids Research.

[42] David C (1972). Regression models and life tables. Journal of the Royal Statistical Society B.

[43] Kaplan EL, Meier P (1958). Nonparametric estimation from incomplete observations. J Amer Statist Assn.

[44] Birte V, Jesper W (2004). LNA (Locked nucleic acid): High-affinity targeting of complementary RNA and DNA. Biochemistry.

[45] Friedman RC, Farh KK, Burge CB, Bartel DP (2009). Most mammalian mRNAs are conserved targets of microRNAs. Genome Res.

[46] Lall S, Grun D, Krek A, Chen K, Wang YL, Dewey CN, Sood P, Colombo T, Bray N, Macmenamin P, Kao HL, Gunsalus KC, Pachter L, Piano F, Rajewsky N (2006). A genome-wide map of conserved microRNA targets in C. elegans. Curr. Biol.

[47] Betel D, Wilson M, Gabow A, Marks DS, Sander C (2008). The microRNA. org resource: targets and expression. Nucleic Acids Res.

