# Supplementary material for: MicroRNA-140-5p targets insulin like growth factor 2 mRNA binding protein 1 (IGF2BP1) to suppress cervical cancer growth and metastasis
Source: Oncotarget. 2016 Aug 31;7(42):68397–411. doi: 10.18632/oncotarget.11722 (PMC5356564; doi:10.18632/oncotarget.11722)
Supplement: Supplementary file 1 [file oncotarget-07-68397-s001.pdf]

MicroRNA-140-5p targets insulin like growth factor 2 mRNA binding protein 1 (IGF2BP1) to suppress cervical cancer growth and metastasis

SUPPLEMENTARY FIGURES

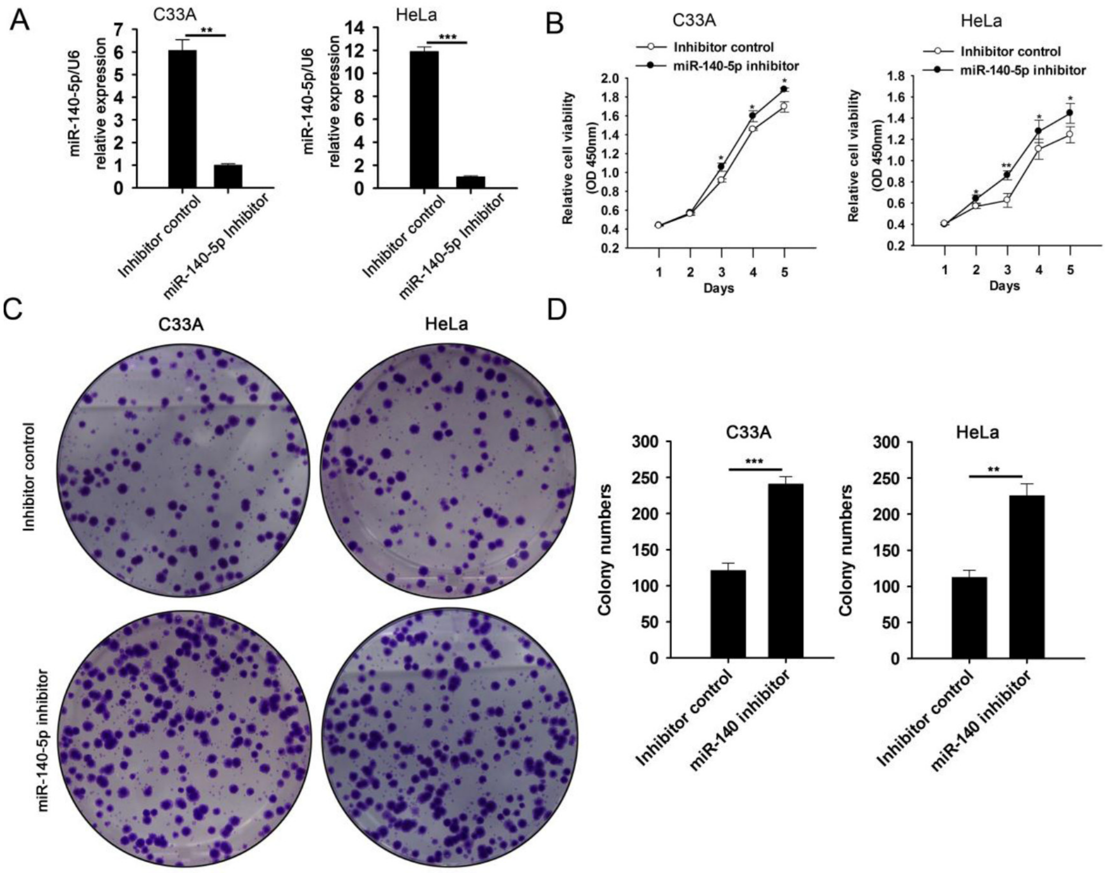

Supplementary Figure S1: Effects of miR-140-5p inhibition on CC cells proliferation in vitro. A. qRT-PCR analysis of miR-140-5p expression in miR-140-5p-inhibitor-transfected and inhibitor-control-transfected C33A and HeLa cells. **B.** CCK8 assays analysis of the effects of miR-140-5p inhibition on cell viability. **C-D.** Colony formation of miR-140-5p-inhibitor transfected C33A and HeLa cells.

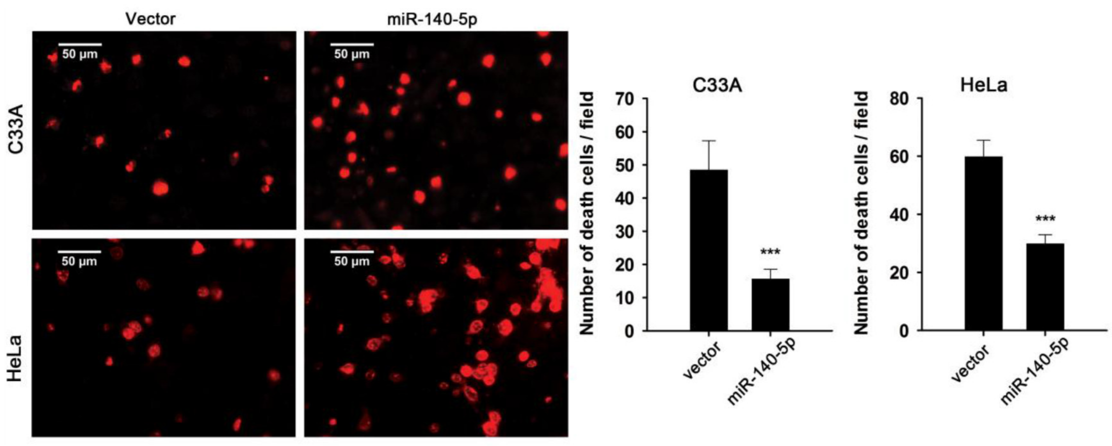

Supplementary Figure S2: miR-140-5p over-expression suppresses CC cell death in vitro.

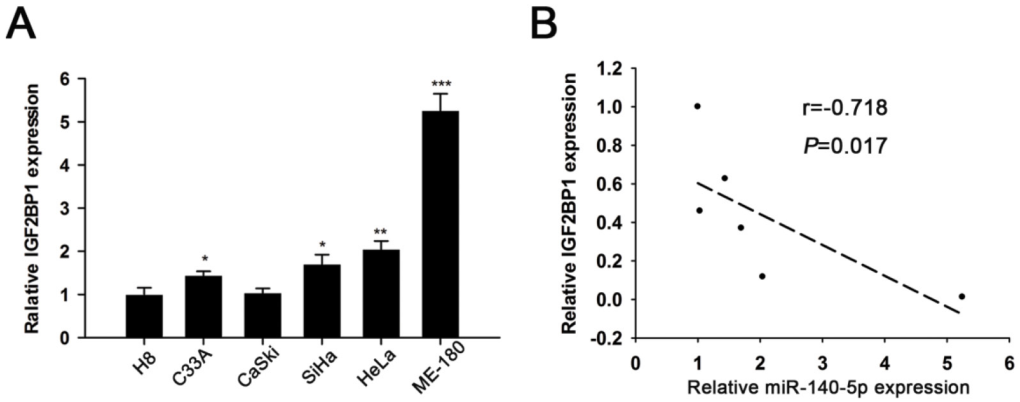

Supplementary Figure S3: A. qRT-PCR analysis of IGF2BP1 mRNA levels in CC cell lines. **B.** Spearman's correlation analysis between miR140-5p levels and IGF2BP1 mRNA levels in cell lines.

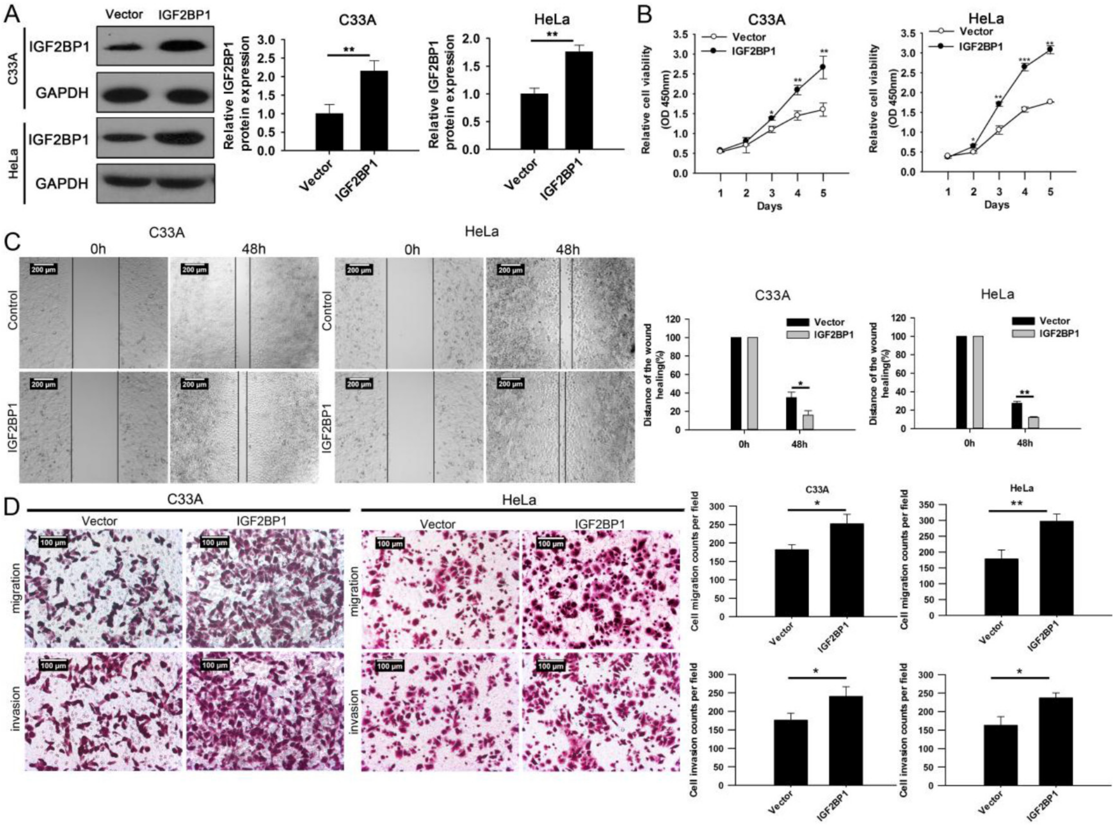

Supplementary Figure S4: IGF2BP1 over-expression promotes CC cell proliferation, migration and invasion in vitro. **A.** Western blot analysis of IGF2BP1 expression in IGF2BP1-siRNA-transfectd or mock-transfected C33A and HeLa cells. **B.** CCK8 assays analysis of the effects of IGF2BP1 over-expression on cell proliferation in C33A and HeLa cells. **C.** Wound healing assays analysis of the effects of IGF2BP1 over-expression on cell migration in C33A and HeLa cells. **D.** Transwell assays analysis of the effects of IGF2BP1 over-expression on cell invasion and migration in C33A and HeLa cells.

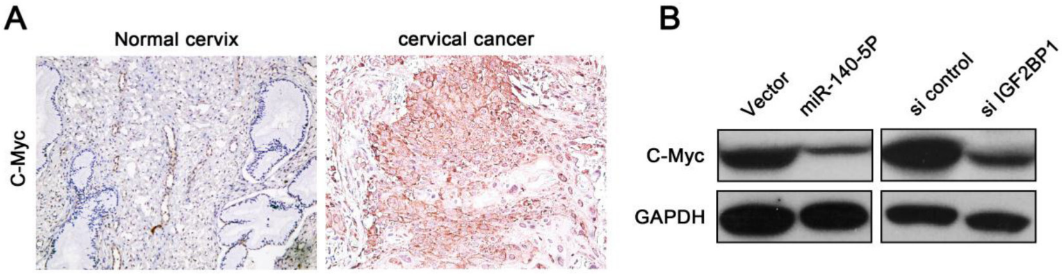

Supplementary Figure S5: The effects of miR-140-5p and IGF2BP1 on c-Myc expression. **A.** IHC analysis of c-Myc expression in nomal cervix and CC tissues. **B.** Western blot analysis of the effects of miR-140-5p and IGF2BP1 down-regulation in C33A cell on c-Myc expression.
